# Phase angle is a predictor for postoperative complications in colorectal cancer

**DOI:** 10.3389/fnut.2024.1446660

**Published:** 2024-08-16

**Authors:** Xiao-Yu Liu, Bing Kang, Quan Lv, Zi-Wei Wang

**Affiliations:** ^1^Department of Gastrointestinal Surgery, the First Affiliated Hospital of Chongqing Medical University, Chongqing, China; ^2^Department of Clinical Nutrition, The First Affiliated Hospital of Chongqing Medical University, Chongqing, China

**Keywords:** colorectal cancer, complications, surgery, phase angle, nomogram

## Abstract

**Aim:**

The aim of this study was to develop a validated nomogram to predict the risk of postoperative complications in colorectal cancer (CRC) patients by analyzing the factors that contribute to these complications.

**Methods:**

We retrospectively collected clinical information on patients who underwent CRC surgery at a single clinical center from January 2021 to December 2021. Univariate and multivariate logistic regression analysis to identify independent risk factors for postoperative complications and to develop a predictive model. A receiver operating characteristic (ROC) curve was used to calculate the area under the curve (AUC) to assess the predicted probability. Calibration curve was drawn to compare the predicted probability of the nomogram with the actual probability, and decision curve analysis (DCA) was employed to evaluate the clinical utility of the nomogram.

**Results:**

A total of 190 CRC patients were included in this study. We retrospectively collected baseline information, clinical information, surgical information, and nutrition-related indicators for all patients. Through multivariate logistic regression analysis, preoperative albumin (*p* = 0.041, OR = 0.906, 95% CI = 0.824–0.996), surgical time (*p* = 0.009, OR = 1.006, 95% CI = 1.001–1.010), waistline (*p* = 0.049, OR = 1.011, 95% CI = 1.002–1.020) and phase angle (PA) (*p* = 0.022, OR = 0.615, 95% CI = 0.405–0.933) were identified as independent risk factors for postoperative complications in CRC, and a nomogram prediction model was established using the above four variables. The AUC of 0.706 for the ROC plot and the high agreement between predicted and actual probabilities in the calibration curves suggested that the prediction model has good predictive power. The DCA also confirmed the good clinical performance of the nomogram.

**Conclusion:**

This study developed a nomogram to predict the risk of postoperative complications in CRC patients, providing surgeons with a reliable reference to personalized patient management in the perioperative period and preoperative nutritional interventions.

## Introduction

According to Global Cancer Data 2020, colorectal cancer (CRC) has become the third most common new cancer diagnosis and the second leading cause of cancer-related deaths worldwide (1–3). In 2020, approximately 1,931,590 people were diagnosed with CRC and 935,173 people died from CRC (4, 5). The World Health Organization (WHO) estimates that by 2030, new cases of CRC will increase by 77% and deaths from CRC will increase by 80% (6, 7). The main treatments for CRC include surgery, local radiotherapy and systemic treatments such as chemotherapy and targeted therapy (8, 9).

Currently, radical surgery remains the cornerstone of CRC treatment (10, 11). Several previous studies have shown that postoperative complications affect the prognosis of CRC patients (12–14). Because CRC affects the intake and absorption of food and nutrients, it often leads to malnutrition in patients (15, 16). Malnutrition can cause changes in immune, respiratory and renal function and impairs wound healing (17, 18). Therefore, comprehensive preoperative assessment of the patient’s nutritional status and timely intervention should receive the full attention of clinicians.

Various objective measures are often used to assess the nutritional status of patients, mainly including anthropometric measures, Subjective Gross Assessment (SGA), Nutritional Risk Score 2002 (NRS2002) and Bioelectrical impedance analysis (BIA) related indicators (19, 20). Anthropometric measurements mainly include body mass index (BMI), waistline, hipline, calf line and triceps skin fold (TSF).

SGA is a nutritional assessment of patients based on subjective patient interviews combined with objective measurement data. Patient-generated SGA (PG-SGA) is an adaptation of SGA and is specifically used as a reference method to assess nutritional status in cancer patients (21, 22). Several studies have shown that PG-SGA is associated with a poorer clinical prognosis in cancer patients (23–25). The PG-SGA primarily assesses the patient’s weight change over a 2-week period, changes in dietary intake, symptoms of nutritional effects, activity and function, and physical examination findings (26).

NRS-2002 is a screening tool for malnutrition, endorsed by the European Society for Clinical Nutrition and Metabolism (ESPEN) for used in hospitalized patients (27, 28). The NRS-2002 is divided into two sections: the patient’s nutritional status and current illness and severity, which are assessed on the basis of the patient’s age, weight loss, body mass index (BMI), food intake and severity of illness (29).

The aim of this study was to collect clinical information and preoperative nutrition-related indicators from CRC patients, analyze the factors leading to postoperative complications, and to establish a valid nomogram to predict the risk of these complications.

## Materials and methods

### Patient selection

We retrospectively collected information on patients who underwent CRC surgery from January 2021 to December 2021 at a single clinical center, including baseline information, clinical information, and surgical information. Inclusion criteria were patients with preoperative pathologically confirmed colorectal malignancy who underwent laparoscopic radical surgery. Exclusion criteria were as follows: 1. Patients who underwent CRC surgery after recurrence; 2. Patients with metastatic CRC; and 3. Patients with incomplete baseline or surgical information. Finally, 190 patients were included.

This study was reviewed by the Ethics Committee of the First Affiliated Hospital of Chongqing Medical University (2022–135-2). It complied with the principles of medical ethics and the Declaration of Helsinki, and all patients participating in the study signed an informed consent form.

### Data elements

We retrospectively collected baseline information, clinical information, surgical information, and nutrition-related indicators from patients. Baseline information included age, sex, BMI, smoking and drinking history, and preoperative comorbidities. Clinical information included preoperative albumin and hemoglobin, tumor location, tumor stage and tumor size. Surgical information included surgical time, blood loss and postoperative complications. Nutrition-related indicators included waistline, hipline, calf line, TSF, NRS2002, PG-SGA and BIA related indicators.

Preoperative comorbidities included hypertension and type 2 diabetes mellitus (T2DM). The tumor was comprehensively staged according to the 8th edition of the AJCC guidelines and was classified as stage I-IV (30).

### BIA

BIA is a non-invasive, practical, objective and simple method of assessing body composition and measuring nutritional status that can be applied to both healthy and patient populations (18, 19, 31–34). BIA uses the measurement of impedance as low amplitude (800 mA) and high frequency (50 kHz) electrical currents pass through the body to calculate indicators of body composition such as muscle, fat, cell mass and volumetric loading status (35, 36).

**Figure 1:**
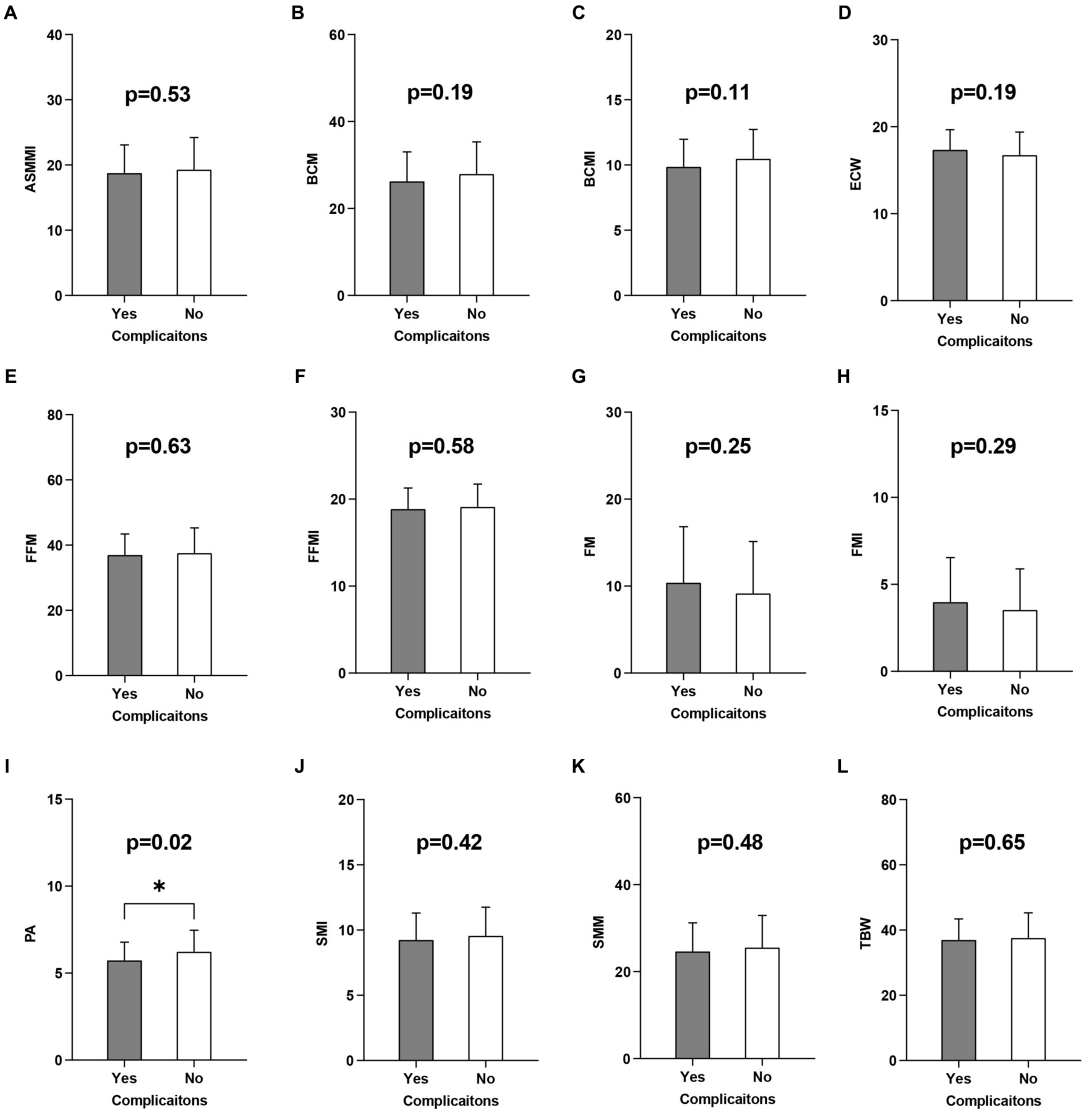
Univariate logistic regression analysis of the BIA related indicators. BIA, Bioelectrical impedance analysis; ASMMI, appendicular skeletal muscle mass index; BCM, body cell mass; BCMI, body cell mass index; ECW, extracellular water; FFM, fat free mass; FFMI, fat free mass index; FM, fat mass; FMI, fat mass index; PA, phase angle; SMI, skeletal muscle index; SMM, skeletal muscle mass; TBW, total body water. **(A-L)** are the results of a univariate logistic analysis of the above indicators of the development of postoperative complications.

BIA is a body composition analysis technique that quantifies various body tissue components and directly measures impedance, phase angle (PA) and other metrics (37, 38). The indices measured by BIA in this study included: appendicular skeletal muscle mass index (ASMMI), body cell mass (BCM), body cell mass index (BCMI), extracellular water (ECW), fat free mass (FFM), fat free mass index (FFMI), fat mass (FM), fat mass index (FMI), PA, skeletal muscle index (SMI), skeletal muscle mass (SMM), total body water (TBW) (Figure 1).

### Statistical analysis

All data in this study were processed using SPSS (version 22.0) and R (version 4.1.2). Continuous variables that followed a normal distribution were expressed as mean ± standard deviation (SD) and comparisons were made using t-test; continuous variables that did not follow a normal distribution were expressed as median and interquartile range (IQR) and assessed using chi-squared or Fisher’s exact test; and categorical variables were expressed as numbers and percentages. Univariate logistic regression analysis was performed on all variables, and variables with *p* < 0.05 were considered potential risk factors for the occurrence of postoperative complications in CRC patients. The screened potential risk factors were then subjected to multivariate logistic regression analysis to identify independent predictors of complications after CRC surgery. Finally, variables with *p* < 0.05 in the multivariate logistic regression analysis were included and a nomogram was created to predict the risk of postoperative complications in CRC.

The predictive model was evaluated in three ways. First, the predictive value of the risk factors was verified using the receiver operating characteristic (ROC) curve, and the area under the curve (AUC) was calculated to assess the performance of the nomogram. The AUC took values between 0 and 1, with 1 indicating perfect agreement, 0.5 indicating no better than chance, and greater than 0.7 indicating that the model has relatively good predictive power (39, 40). Secondly, prediction curves were plotted to test the correction of the postoperative complication risk map, comparing the predicted and actual probabilities of the nomogram, using the 45 degree line as a perfect model with 100% accuracy (41). Finally, decision curve analysis (DCA) was used to analyze the net benefit based on different threshold probabilities to determine the clinical applicability of the nomogram (42, 43).

## Results

### Baseline information

Based on the above inclusion and exclusion criteria, a total of 190 patients who underwent CRC surgery were included in this study. This included 42 patients in the complication group and 148 patients in the no complication group. Comparing the baseline information of the two groups of patients, we found a statistical difference (*p* < 0.05), including preoperative albumin (*p* = 0.005), surgical time (*p* = 0.004), blood loss (*p* = 0.010), and waistline (*p* = 0.043; Table 1).

**Table 1:** Baseline information between the complications group and no complications group.

Characteristics	Complications (42)	No complications (148)	*p* value
Age, year	64.9 ± 13.4	61.1 ± 12.1	0.083
Sex			
Male	28 (66.7%)	88 (59.5%)	0.398
Female	14 (33.3%)	60 (40.5%)	
BMI, kg/m^2^	22.6 ± 3.2	22.6 ± 3.2	0.277
Smoking	18 (42.9%)	59 (39.9%)	0.727
Drinking	17 (40.5%)	59 (39.9%)	0.943
Hypertension	14 (33.3%)	36 (24.3%)	0.242
T2DM	9 (21.4%)	23 (15.5%)	0.368
Albumin, g/L	39.4 ± 4.2	41.4 ± 4.1	0.005*
Hemoglobin, g/L	117.3 ± 25.7	121.4 ± 21.9	0.306
Tumor location			0.572
Colon	15 (35.7%)	60 (40.5%)	
Rectum	27 (64.3%)	88 (59.5%)	
TNM stage			0.394
I	4 (9.5%)	20 (13.5%)	
II	9 (21.5%)	30 (20.3%)	
III	19 (45.2%)	78 (52.7%)	
IV	10 (23.8%)	20 (13.5%)	
Tumor size			0.231
< 5 cm	31 (73.8%)	122 (82.4%)	
≥ 5 cm	11 (26.2%)	26 (17.6%)	
Surgical time, min	214.0 ± 109.8	177.3 ± 57.6	0.004*
Blood loss, mL	180.0 ± 559.2	55.4 ± 96.7	0.010*
Waistline, cm	83.2 ± 8.0	80.1 ± 8.9	0.043*
Hipline, cm	91.2 ± 5.8	89.4 ± 6.3	0.095
Calf line, cm	31.2 ± 3.1	31.5 ± 3.5	0.529
TSF, mm	15.0 ± 6.2	13.5 ± 6.0	0.159
PG-SGA	4.0 ± 2.1	4.5 ± 2.5	0.174
NRS2002	2.1 ± 1.1	2.1 ± 1.0	0.878

Variables are expressed as the mean ± SD, *n* (%), *p-value < 0.05. BMI, body mass index; T2DM, type 2 diabetes mellitus; TSF, triceps skin fold; PG-SGA, Scored Patient-Generated Subjective Global Assessment; NRS2002, nutrition risk screening 2002.

### Nomogram variable screening

Univariate and multivariate logistic regression analysis were performed on a total of 32 potential factors, including baseline information, clinical information, surgical information, and nutrition-related indicators, to find the risk factors affecting the occurrence of complications after CRC surgery. The results of univariate logistic regression analysis showed that preoperative albumin (*p* = 0.005, OR = 0.879, 95% CI = 0.804–0.962), surgical time (*p* = 0.009, OR = 1.006, 95% CI = 1.001–1.010), waistline (*p* = 0.046, OR = 1.042, 95% CI = 1.001–1.084) and PA (*p* = 0.016, OR = 0.622, 95% CI = 0.424–0.914) were the potential risk factors for postoperative complications of CRC (Table 1; Figure 1). Further multivariate logistic regression analysis of the four potential risk factors showed that preoperative albumin (*p* = 0.041, OR = 0.906, 95% CI = 0.824–0.996), surgical time (*p* = 0.009, OR = 1.006, 95% CI = 1.001–1.010), waistline (*p* = 0.049, OR = 1.011, 95% CI = 1.002–1.020) and PA (*p* = 0.022, OR = 0.615, 95% CI = 0.405–0.933) were independent risk factors for the occurrence of postoperative complications in CRC (Table 2).

**Table 2:** Multivariate logistic regression analysis of the complications.

Risk factors	Multivariate logistic regression analysis	
	OR (95% CI)	*P* value
Albumin, g/L	0.906 (0.824–0.996)	0.041*
Surgical time, min	1.006 (1.001–1.010)	0.009*
Waistline, cm	1.011 (1.002–1.020)	0.049*
PA	0.615 (0.405–0.933)	0.022*

*P-value < 0.05. OR, odds ratio; CI, confidence interval, PA, Phase angle.

### Nomogram construction and validation

**Figure 2:**
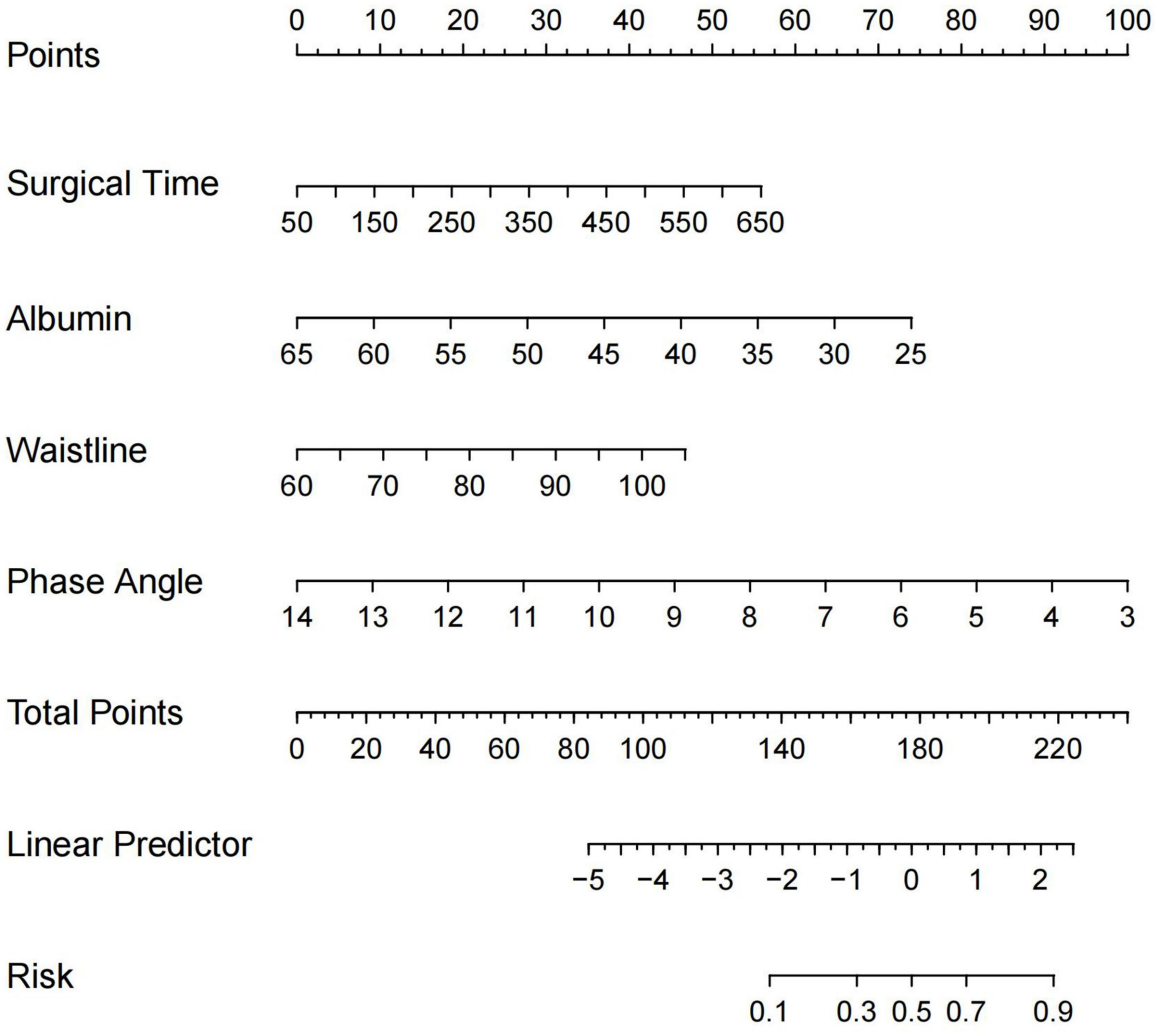
Nomogram for predicting the risk of postoperative complications after CRC surgery. CRC, colorectal cancer.

A nomogram model for predicting the risk of postoperative complications in CRC patients was constructed using four independent risk factors identified by multivariate logistic regression analysis. As shown in Figure 2, the corresponding scores of each factor were obtained according to the patients’ own actual situation, and the total score was obtained by adding the four scores, and the final predicted risk of postoperative complications was the probability corresponding to an individual patient’s total score.

**Figure 3:**
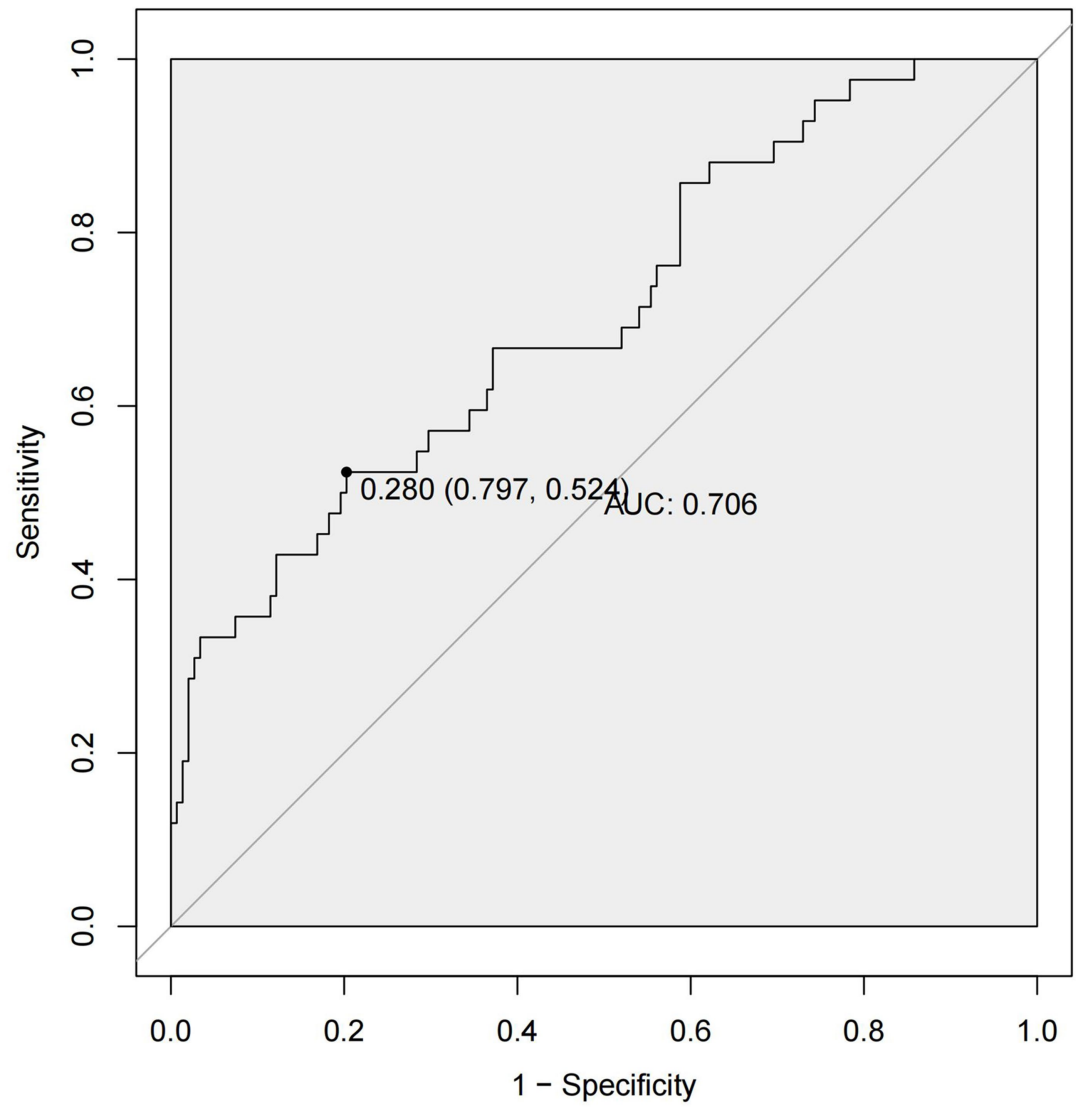
ROC curves of the nomogram. ROC, receiver operating characteristic; AUC, area under the curve.

**Figure 4:**
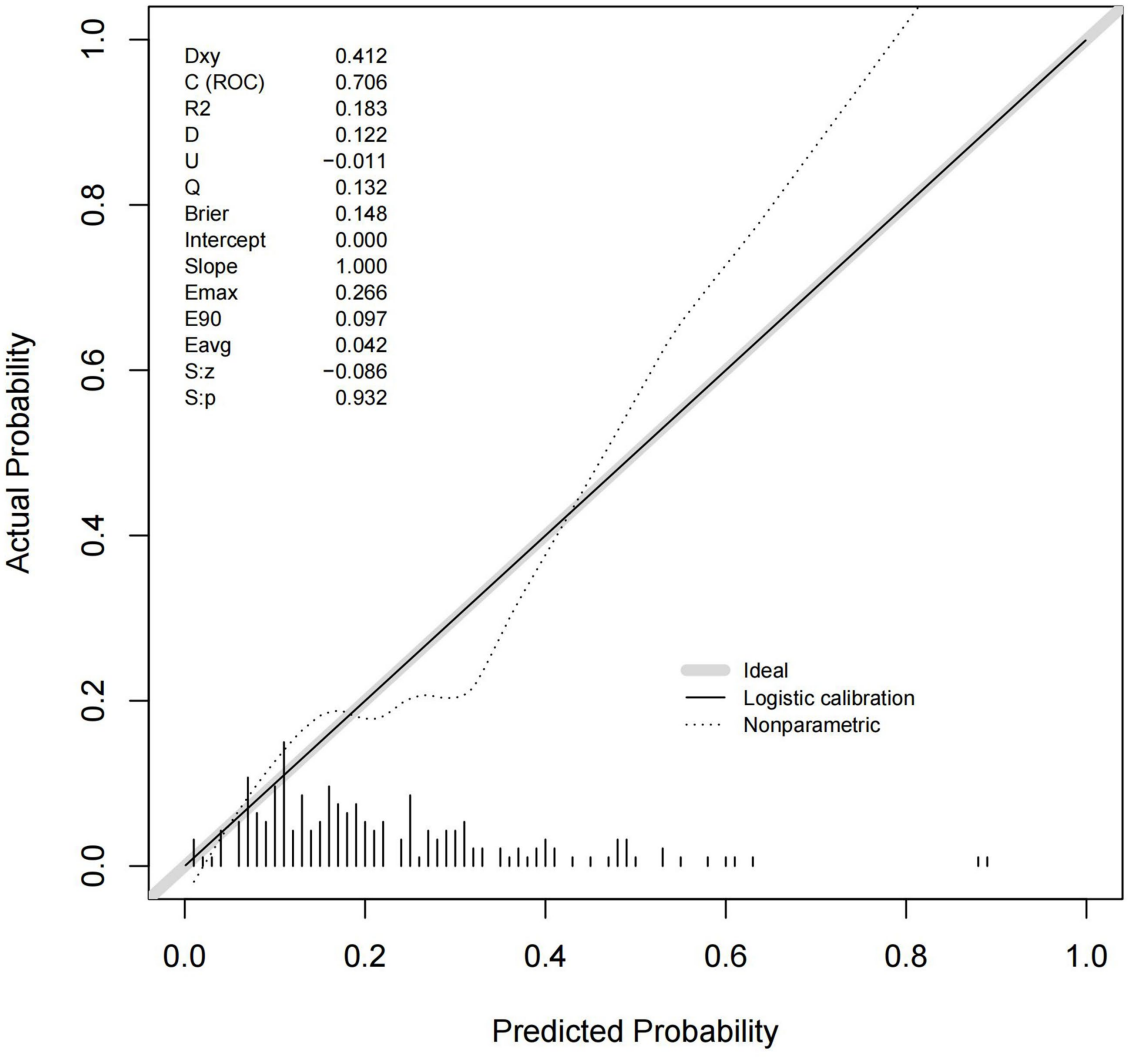
Calibration curve for the nomogram.

**Figure 5:**
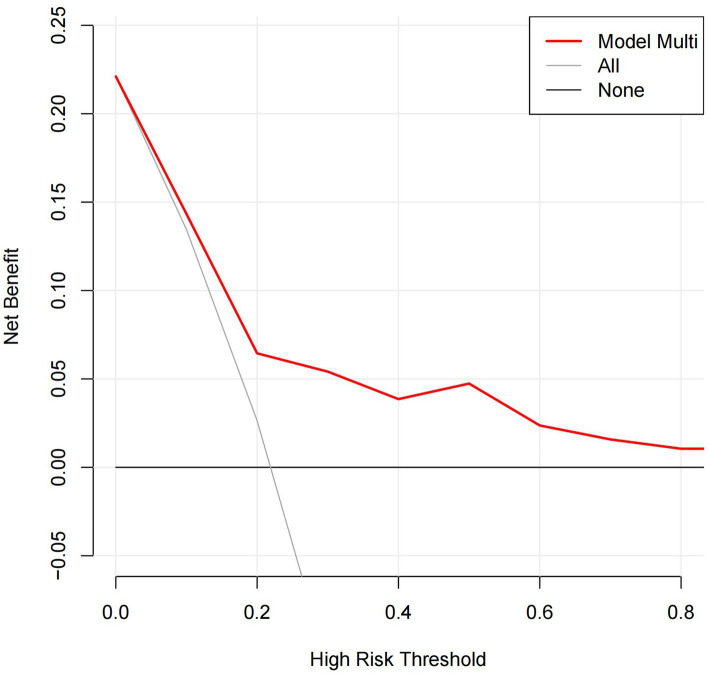
DCA for the nomogram. DCA, decision curve analysis.

By ROC analysis, AUC of the nomogram over time was 0.706, indicating that the model has good predictive performance (Figure 3). The calibration curve showed that there was a high degree of agreement between the predicted and observed results of the nomogram model constructed in this study (Figure 4). Finally, DCA was used to evaluate the clinical application value of the prediction model, as shown in Figure 5.

## Discussion

A total of 190 patients undergoing CRC surgery were included in this study, including 42 patients in the complication group and 148 patients in the no complication group. Multivariate logistic regression analysis revealed that preoperative albumin, surgical time, waistline and PA were independent risk factors for postoperative complications of CRC. Using these four independent risk factors, we constructed a nomogram model to predict the risk of postoperative complications in CRC patients.

The results of this study suggested that lower preoperative albumin, longer surgical time, larger waistline and smaller PA were independent risk factors for postoperative complications in CRC. Several previous studies have shown that low preoperative albumin levels were a risk factor for postoperative complications and survival in several types of cancer, including CRC (44–47). This might be because low preoperative albumin was a marker of malnutrition, cancer cachexia and chronic inflammatory activity, among other factors (48, 49).

The results of the multivariate logistic regression analysis showed that the duration of surgery was an independent risk factor for the development of postoperative complications in CRC, which was consistent with previous literature (50, 51). Prolonged surgical time not only increased tissue contraction, causing tissue ischaemia, desiccation and necrosis, but also led to coagulation, hemostasis and endothelial damage, thereby increasing the risk of venous thromboembolism (52, 53). In addition, the effectiveness of antimicrobial prophylaxis decreased over time and the likelihood of aseptic technique violations increases (54). Longer surgical time also meant longer anesthesia time and increased fatigue for the surgical team (55).

Waistline was an indicator of central obesity and was often considered a more useful perioperative risk assessment tool than BMI in CRC surgery (56, 57). Increased waistline affected the incidence of postoperative complications in CRC, which might be due to prolonged surgical time, relative wound hypoxia due to reduced subcutaneous tissue oxygenation, impaired collagen synthesis and immune system function (58, 59). Moreover, abdominal obesity was often associated with high levels of inflammatory mediators such as interleukin-6, c-reactive protein and tumor necrosis factor-α, which often led to insulin resistance and postoperative complications (60, 61).

PA was a BIA correlated that was directly related to the quality and integrity of cell membranes, and was often considered a marker of cellular health (62, 63). PA was closely related to the level of inflammation in the body, and inflammation and low albumin were associated with altered fluid distribution in the extracellular space, which could reduce the PA (64, 65). In addition, PA was a marker of oxidative stress, whereby an imbalance between oxidants and antioxidants produced oxidative stress, leading to the destruction of cellular components such as proteins or lipids, which could lead to cellular damage and rupture of cell membranes (65–67).

However, this study still has some limitations. First, this was a single-center retrospective study. Secondly, the nomogram prediction model developed in this study was not internally validated, and we will continue to collect clinical information and nutritional indicators from the patients concerned to improve internal validation. The application of deep learning algorithms has the potential to improve the accuracy and effectiveness of CRC detection (68, 69), however, due to the small sample size of this study, the application of deep learning algorithms was not carried out and it is expected that in the future appropriate validation can be carried out when large data sets are available.

In conclusion, this study created a nomogram to predict the risk of postoperative complications in CRC patients, providing surgeons with a reliable reference to personalized patient management in the perioperative period and preoperative nutritional interventions.

## Data Availability

The raw data supporting the conclusions of this article will be made available by the authors, without undue reservation.
